# Sense identification data: A dataset for lexical semantics

**DOI:** 10.1016/j.dib.2020.106267

**Published:** 2020-09-03

**Authors:** Davide Colla, Enrico Mensa, Daniele P. Radicioni

**Affiliations:** Computer Science Department, University of Turin, Italy

**Keywords:** Sense annotation, Sense individuation, Lexical processing, Lexical semantics, Word embeddings, Sense embeddings, Semantic similarity, Similarity metrics

## Abstract

Sense Identification is a newly proposed task; in considering a pair of terms to assess their conceptual similarity, human raters are postulated to preliminarily select a sense pair. Senses involved in this pair are those actually subject to similarity rating. The sense identification task is searching for the sense selected during the similarity rating. The sense individuation task is important to investigate strategies and sense inventories underlying human lexical access and, moreover, it is a relevant complement to the semantic similarity task. Individuating which senses are involved in the similarity rating is also crucial in order to fully assess those ratings: if we have no idea of which two senses were retrieved, on which base can we assess the score expressing their semantic proximity?

The Sense Identification Dataset (SID) dataset has been built to provide a common experimental ground to systems and approaches dealing with the sense identification task. It is the first dataset specifically designed for experimenting on the mentioned task. The SID dataset was created by manually annotating with sense identifiers the term pairs from an existing dataset, the SemEval-2017 Task 2 English dataset. The original dataset was originally conceived for experimenting on the semantic similarity task, and it contains a score expressing the human similarity rating for each term pair. For each such term pair we added a pair of annotated senses: in particular, senses were annotated such that they are compatible (explicative of) with the existing similarity ratings. The SID dataset contains BabelNet sense identifiers. This sense inventory is a broadly adopted ‘naming convention’ for word senses, and such identifiers can be easily mapped onto further resources such as WordNet and WikiData, thereby enabling further processing tasks and usages in the Natural Language Processing pipeline.

## Specifications Table

**Table:** 

Subject	Artificial Intelligence
Specific subject area	Data related to Natural language processing (NLP) tasks, and specifically intended for Lexical Semantics experiments.
Type of data	Table Tab-Separated-Value (TSV)
How data were acquired	The dataset has been obtained by annotating with word senses a list of word pairs, each pair being equipped with a similarity score. The term pairs along with their similarity score were presented to three fluent English speakers. The annotators were asked to provide word senses by considering both the term pair and the provided similarity score.
Data format	Raw data in textual (tsv: tab separated values) format.
Parameters for data collection	All senses were collected by querying BabelNet [3] with each term in pairs.
Description of data collection	Overall 15,558 senses were collected for the 1,000 lexical items; a script was developed in order to simplify the annotation task, illustrating all available senses for both terms in each pair.
Data source location	The original dataset was compiled at the University of Roma La Sapienza, while sense identification data was collected at the University of Turin, Department of Computer Science. The dataset is hosted on Mendeley Data, and available at the URL http://dx.doi.org/10.17632/r5fbdpvnkk.1.
Data accessibility	Repository name: Sense Identification Dataset - SID Data identification number: Colla, Davide; Mensa, Enrico; Radicioni, Daniele P. (2020), “Sense Identification Dataset - SID”, Mendeley Data, v1 http://dx.doi.org/10.17632/r5fbdpvnkk.1 Direct URL to data:https://data.mendeley.com/datasets/r5fbdpvnkk/1
Related research article	D. Colla, E. Mensa and D. P. Radicioni, Novel Metrics for Computing Semantic Similarity with Sense Embeddings, accepted for publication in the *Knowledge-Based Systems* journal, https://doi.org/10.1016/j.knosys.2020.106346.

## Value of the Data

• As illustrated in [1], the task of semantic similarity should be complemented by the task of sense individuation. While various datasets exist that were devised to experiment on the semantic similarity task, the SID dataset allows experimenting on both the semantic similarity task and on the sense identification task.
• Researchers working on word and sense embeddings have a scientific base for assessing the representational precision of their embeddings and the appropriateness of their strategy in individuating which senses are involved in the semantic similarity task.
• The dataset provides a starting point for exploring how human similarity rating actually works. Cognitive strategies, algorithms and systems dealing with semantic similarity, sense embeddings and new similarity metrics should be tested on the SID dataset. Their performances in both tasks can be compared against those provided in [1], obtained by experimenting with six recent and influential sets of embeddings, namely LessLex [1], NASARI [7], DeConf [8], SenseEmbed [9], SW2V [10], and LSTMEmbed [11].

## 1 Data Description

The dataset consists of 500 word pairs along with their semantic similarity scores and annotated senses. All records are contained into a single tab separated file, and each such entry has the following fields: *word1, word2*, semantic similarity score, senses for *word1* and senses for *word2*.

The Sense Identification dataset (SID) builds on previous data, originally conceived for the international competition SemEval 2017 Task 2 [2].^1^ Namely, each entry was originally composed by a triple 〈*t, u, y*〉 containing the term pair *t, u* and a numeric score *y* expressing the similarity between the considered terms.

Each such tuple has now been extended by adding two sets of senses, *S_t_* and *S_u_*, containing the BabelNet synsets [3] for *t* and *u*. Borrowing the sense inventory and the sense identifiers from BabelNet as our naming convention allows to directly link the SID dataset to resources such as WordNet [4], DBPedia [5], and Wikidata [6]. The annotated senses have been chosen as appropriate for the terms at stake and compatible with the similarity score *y*.

The annotation consisted in selecting the most appropriate senses for each term pair 〈*t, u*〉. As an example, in Table 1 we report some of the senses for the word pair *<cinnamon, candy>*. The most prominent sense for each term at stake was selected by considering the other term as a minimal though effective disambiguation context; this salience criterion amounts to computing the argument maximizing the semantic similarity between all sense pairs, as illustrated in [1]. In the end the sets of annotated senses are *S_cinnamon_* = {bn:00019142n; bn:00019141n} and *S_candy_* = {bn:00015227n}. It is worth noting that *S* is a set rather than a single sense since multiple instances of overlapping senses can be detected in the BabelNet sense inventory, but each *S* only contains *equivalent* senses. For example, the term *cinnamon* has been annotated with the two senses corresponding to 'Spice from the dried aromatic bark of the Ceylon cinnamon tree; used as rolled strips or ground' (bn:00019142n) and 'Aromatic bark used as a spice' (bn:00019141n). On average over all term pairs, each term was annotated with 1.09 senses.

**Table 1:** List of senses for the word pairs.

Senses of 'Cinnamon' (BabelNet synset ID; text description)	Senses of 'Candy' (BabelNet synset ID; text description)
bn:00017431n; Tropical Asian tree with aromatic yellowish-brown bark	bn:00015227n; A rich sweet made of flavored sugar and often combined with fruit or nuts
bn:00019141n; Aromatic bark used as a spice	bn:03066256n; "Candy" is a song by English pop singer, Robbie Williams
bn:00019142n; Spice from the dried aromatic bark of the Ceylon cinnamon tree	bn:01857133n; Candy is a 2006 Australian romantic drama film
bn:17156147n; Cinnamon is a free and open-source desktop environment for the X Window System that derives from GNOME 3	bn:03121020n; Candy is a large family-owned Italian company based in Brugherio, near Milan, that manufactures domestic appliances.
bn:04953402n; Color	
bn:02202236n; "Cinnamon" is a song by American rock band Stone Temple Pilots.	

## 2 Experimental Design, Materials and Methods

Out of the 500 starting pairs we dropped 8 pairs, thereby resulting in a grand total of 492 annotated pairs. For the 984 terms therein, overall 15,558 Babel synsets were found, corresponding to 144,262 possible sense combinations, on average over 293 per term pair. Such annotated data is featured by averaged pairwise 0.89 Cohen's *k* inter annotator agreement on the individual terms, and 0.79 on term pairs.

We started from a recent dataset, the SemEval-2017 Task 2 — Subtask 1 English dataset [2]. The original 500 word pairs (all of them nouns, that include named entities) were annotated with a similarity score. In order to collect the 500 English term pairs, the authors chose 34 domains from the BabelNet semantic network: from each domain 12 words were sampled, requiring at least one multi-word expression and two named entities to be included. In order to pick up words possibly out of any pre-defined domain, the authors added 92 extra words, whose domain was not decided beforehand. Given the set of the initial 500 seed words, the pairs were generated so to ensure a uniform distribution of pairs across the similarity scale. The similarity scores featuring each term pair are based on a five-point Likert scale —ranging from 0, which means “totally dissimilar and unrelated'' to 4, which stands for “very similar''—. Rating criteria used by SemEval 2017 annotators are reported in Table 2.

**Table 2:** Annotation guidelines adopted in the SemEval 2017 dataset.

Score	Description	
0	Totally dissimilar and unrelated	The two words do not mean the same thing and are not on the same topic (e.g., pencil-frog or PlayStation- monarchy).
1	Dissimilar	The two words describe clearly dissimilar concepts, but may share some small details, a far relationship or a domain in common and might be likely to be found together in a longer document on the same topic (e.g., software-keyboard or driver-suspension).
2	Slightly similar	The two words do not have a very similar meaning, but share a common topic/domain/function and ideas or concepts that are related (e.g., house-window or airplane-pilot)
3	Similar	The two words share many of the important ideas of their meaning but include slightly different details. They refer to similar but not identical concepts (e.g., lion-zebra or firefighter-policeman).
4	Very similar	The two words are synonyms (e.g., midday-noon or motherboard-mainboard).

The original contribution in the SID dataset consists in adding to such data the annotation on the senses possibly underlying the word pairs in the considered dataset. Three researchers fluent in English were recruited to annotate the 500 word pairs. The annotated senses had to be coherent with the word pair *t, u* and with the similarity score *y*. For example, given the word pair *<fault, system>* and the associated score 0.58, the annotators were requested to indicate the identifiers corresponding to senses that justified the score 0.58, indicating a low degree of similarity, the following senses were selected: the sense of fault as ‘An imperfection in an object or machine’, and the sense of system as ‘Instrumentality that combines interrelated interacting artifacts designed to work as a coherent entity’ (corresponding to the Babel synset identifiers bn:00025865n and bn:00075759n, respectively). If, on the other hand, the existing similarity rating had been higher, one could have expected that the annotators would have chosen closer (and more similar) senses, such as those implied in system faults in the computer science domain (e.g., those dealing with hardware exceptions).

In order to simplify the annotation task, a script was developed to query BabelNet, listing all available senses for both terms in each pair. Overall 15,558 senses were collected for the 1000 lexical items in the dataset.

The collected annotations for *S_t_* and *S_u_* were then merged through a simple voting strategy: we chose the senses selected by at least two annotators (*minimal consensus*). Alternatively, if no sense was found in BabelNet for either term, or no minimal consensus was reached on either term, the pair was dropped. Out of the 500 starting pairs we dropped 8 pairs, thereby resulting in a grand total of 492 annotated pairs.

## Ethics Statement

This dataset has been collected with the support of subjects that accepted to join our experiments, after an appropriate information and training phase and by signing an Informed Consent form.

## Declaration of Competing Interest

The authors declare that they have no known competing financial interests or personal relationships which have, or could be perceived to have, influenced the work reported in this article.

## Footnotes

1 Original data available at the URL http://alt.qcri.org/semeval2017/task2/.
